# Socio-demographic determinants of breastfeeding initiation and duration in US children: an analysis of NHANES (1999–2018)

**DOI:** 10.1186/s13006-025-00803-8

**Published:** 2025-12-30

**Authors:** Chaochao Wen, Chutian Shi, Angela Vinturache, Guodong Ding, Yongjun Zhang

**Affiliations:** 1 Department of Pediatrics, Xinhua Hospital, Shanghai Jiao Tong University School of Medicine, 1665 Kongjiang Road, Shanghai, 200092 China; 2 Department of Obstetrics & Gynecology, University of Alberta, Edmonton, Alberta Canada; 3 Department of Neuroscience, University of Lethbridge, Lethbridge, Alberta Canada; 4 Ministry of Education–Shanghai Key Laboratory of Children’s Environmental Health , Xinhua Hospital, Shanghai Jiao Tong University School of Medicine, Shanghai, China

**Keywords:** Socio-demographic factors, Breastfeeding initiation, Breastfeeding duration, Children

## Abstract

**Background:**

Despite an increase in the prevalence of breastfeeding initiation and breastfeeding duration over the past decades in the US, breastfeeding practices remain suboptimal and fall well below the Healthy People 2030 goals. We aimed to investigate the potential associations between socio-demographic factors and breastfeeding initiation and duration, with the goal of identifying factors that act as barriers or facilitators to breastfeeding practices.

**Methods:**

A total of 7,629 US children aged 0 to 24 months were included from the National Health and Nutrition Examination Survey data between 1999 and 2018. Logistic regression was used to examine the relationship between socio-demographic factors and breastfeeding initiation and duration, with a heat map illustrating their combined associations. Meta-regression was employed to estimate the temporal trends in odds ratios (OR) for the effect of socio-demographic factors on breastfeeding outcomes.

**Results:**

The overall prevalence of breastfeeding initiation and duration at 3, 6, and 12 months were lower among non-Hispanic Black children [adjusted OR (aOR): 0.42; 95% confidence interval (CI): 0.35, 0.51; 0.77; 0.63, 0.95; 0.58; 0.47, 0.71; 0.45; 0.32, 0.62] when compared with White children, while higher among high-income families (1.53; 1.31, 1.78; 1.24; 1.04, 1.48; 1.36; 1.15, 1.61; 1.23; 0.96, 1.59), mothers older than 25 years at delivery (1.68; 1.45, 1.95; 1.95; 1.63, 2.34; 1.88; 1.58, 2.23; 1.62; 1.26, 2.08), and nonsmoking mothers during pregnancy (2.65; 2.19, 3.21; 2.10; 1.60, 2.75; 2.52; 1.86, 3.41; 1.97; 1.26, 3.07) than among low-income families, mothers aged ≤ 25 years, and smoking mothers, respectively. When stratified by 10 consecutive cycles, the disparities in temporal trends among most socio-demographic factors remained stable, but the gaps among smoking status even widened over time. Heat map analysis further depicted the combined associations between these factors and breastfeeding practices, and found that children born to Black mothers, smoking mothers, mothers aged ≤ 25 years, and low-income families generally had lower odds of breastfeeding initiation and breastfeeding duration at 3, 6, and 12 months.

**Conclusions:**

Children born to socioeconomically disadvantaged families generally exhibited poorer breastfeeding practices.

**Clinical trial number:**

Not applicable.

## Background

Breastfeeding and human milk provide optimal infant feeding and nutrition, and confer a range of benefits to children, mothers, and society. Children who are breastfed are at reduced risk for otitis media, respiratory tract infections, gastroenteritis, asthma, and obesity, while mothers who breastfeed are at lower risk for postpartum haemorrhage and depression, breast and ovarian cancer, and type 2 diabetes [1]. Scaling up breastfeeding could prevent an estimated 823,000 child deaths and 20,000 maternal deaths globally each year, along with economic savings of US$300 billion [1]. The World Health Organization (WHO) and American Academy of Pediatrics (AAP) recommend exclusive breastfeeding for the first 6 months of life and continued breastfeeding along with appropriate complementary foods for up to 2 years or beyond [2, 3].

In the US, decades of ongoing national breastfeeding promotion efforts have been successful in steadily increasing breastfeeding rates. Among the US National Health and Nutrition Examination Survey (NHAHES) 2017–2018 data, breastfeeding initiation has risen to 87.6%, achieving the Healthy People 2020 national goal of 81.9%, while national rates of continued breastfeeding at 6 (48.2%) and 12 months (21.7%) fell well below public health recommendations of 60.6% and 34.1%, respectively [4, 5]. Although the majority of US mothers begin breastfeeding, the current low rate of breastfeeding continuation suggests that most US infants and mothers are not receiving the maximum health benefits associated with full and extended breastfeeding. The new Healthy People 2030 goals reflect more ambitious targets for all breastfeeding outcome measures, with the proportions of exclusively breastfeed for 6 months increasing to 42.4% and breastfeeding continuation for 12 months to 54.1% [6]. Therefore, increasing breastfeeding rates throughout the US and promoting optimal breastfeeding practices toward the ultimate goal of improving the public’s health remain a critical public health strategy.

There are large breastfeeding disparities between and within countries by socioeconomic characteristics, geographical and cultural context, healthcare system and related health professionals, as well as mother’s attitudes and health status. Socio-demographic factors seem to be very important determinants for breastfeeding practices. Factors such as race and ethnicity, maternal age, socioeconomic status, and smoking habits can significantly impact a mother’s ability and decision to breastfeed. For example, lower socioeconomic status often limits access to healthcare and lactation support, while both younger and older maternal ages may present unique challenges and decisions regarding breastfeeding. Furthermore, different racial and ethnic groups may experience varying social, economic, and cultural pressures, as well as differences in healthcare access, which contribute to disparities in breastfeeding behaviors. Smoking not only poses risks to the mother’s health but can also negatively affect breastfeeding outcomes by influencing maternal behavior and decision-making [7, 8]. A deeper exploration of these contextual factors would provide a more comprehensive understanding of the complexities that shape breastfeeding practices.

Recent studies, using the US National Immunization Survey data, have found that despite significant improvement in breastfeeding initiation and duration rates among black infants during the past decade, racial and ethnic disparities between black and white infants have persisted and the gaps even widened over time [9, 10]. Previous studies also reported that breastfeeding rates were generally lower among infants of mothers who were young, unmarried, less educated, smoking, had lower incomes, or living in rural areas [7, 8]. However, most of these studies were conducted with populations outside US [11] or in specific population subsamples within US [12] rather than a population-based sample. While nationally representative data on breastfeeding behavior exists, much of it is outdated and may not fully reflect the socio-demographic factors influencing current breastfeeding practices [13, 14]. In light of ongoing national campaigns to promote breastfeeding in the US, this study fills a significant gap in the literature by providing current, nationally representative data to investigate both the individual and combined influences of socio-demographic factors that act as barriers or facilitators to breastfeeding behavior, and how these effects evolve over time.

## Methods

NHANES is a program of studies that includes a series of cross-sectional surveys using complex, multistage, stratified, clustered probability design to sample the noninstitutionalized civilian resident US population. Details on the study design, protocol, and data collection methods have been documented [15]. The NHANES was approved by the Centers for Disease Control and Prevention/National Center for Health Statistics Research Ethics Review Board and written parental informed consent was obtained for all infants and toddlers. This study used data across 10 recent cycles of NHANES (1999–2000 through 2017–2018) to estimate the individual and combined associations of socio-demographic characteristics with breastfeeding practice among children aged 0 to 24 months, with response rates ranging from 62.0% to 93.0% [16]. Because publicly available, anonymized data were used, the institutional review board of Xinhua Hospital, Shanghai Jiao Tong University School of Medicine deemed the study exempt from review. This study followed the STROBE reporting guideline.

Among a total of 7639 participants aged 0 to 24 months from 1999 to 2000 to 2017–2018, 10 with missing information on breastfeeding initiation were excluded, resulting in breastfeeding initiation analyses of 7629 participants (51.6% boys, 48.4% girls, 32.1% non-Hispanic White, 19.9% non-Hispanic Black, 39.5% Hispanic, 8.5% other). We further excluded 2060 participants never breastfed or who were breastfeeding but aged younger than 3 (*n* = 608), 6 (*n* = 1035), and 12 (*n* = 1566) months, leaving 4961, 4534, and 4003 participants for breastfeeding duration at 3-, 6-, and 12-month analyses, respectively.

Breastfeeding initiation was defined based on answers to the question “Was the survey participant (SP) ever breastfed or fed breastmilk?”. Breastfeeding duration was defined based on answers to the question “How old was (SP) when (he or she) completely stopped breastfeeding or being fed breast milk?”. Weighted data were used to examine the influence of selected socio-demographic factors including race and ethnicity, poverty index ratio (PIR), maternal age at delivery, and smoking status during pregnancy on breastfeeding initiation and breastfeeding duration at 3, 6, and 12 months. In this study, race and ethnicity was divided into four categories: Hispanic, non-Hispanic White, non-Hispanic Black, and other. PIR was calculated by dividing family income by the poverty guidelines specific to the survey year and was categorized into < 1.30 (low income) and ≥ 1.30 (high income). A PIR value of 1.30 corresponds to a family income 130% of the poverty level for that survey year. Maternal age at delivery was categorized into ≤ 25 years and > 25 years. Smoking status during pregnancy was defined based on answers to the question “Did (SP) biological mother smoke at any time while she was pregnant with the survey participant?”.

Data analysis was performed between 22 April and 24 October 2024. Descriptive statistics including frequency and percentage were used to present the characteristics of the study population, overall and by socio-demographic subgroups. Prevalence of breastfeeding initiation and breastfeeding duration at 3, 6, and 12 months were weighted to be nationally representative, considering the complex survey sampling design of the NHANES. Since all variables in this study were collected through in-home interviews, we applied the appropriate interview weights for analysis, in accordance with the NHANES weighting guidelines [17]. Separate analyses were undertaken for each breastfeeding outcome. Survey-weighted logistic regression model was used to compare and identify the differences of breastfeeding initiation and breastfeeding duration among these socio-demographic subgroups. Confounders were chosen for inclusion in adjusted models based on previous literature: race and ethnicity, PIR, maternal age at delivery, and smoking status during pregnancy [4, 7].

For these significant socio-demographic factors including race and ethnicity, PIR, maternal age at delivery, and smoking status during pregnancy by logistic regression, we further examined the combined associations of these socio-demographic factors with each breastfeeding outcome, using a 4 × 2 × 2 × 2 heat map. In addition, weighted meta-regression was employed to estimate the temporal trends in odds ratios (OR) for the effect of socio-demographic factors on breastfeeding outcomes. Specifically, survey-weighted logistic regressions were performed for each NHANES cycle to obtain cycle-specific log (OR) and standard errors (SE). These estimates were subsequently analyzed using a study-level random-effects meta-regression with inverse-variance weighting. A 2-tailed *p* < 0.05 was considered to be statistically significant, and all statistical analyses were conducted using R Statistical Software (version 4.2.3).

## Results

Table 1 showed the characteristics of the study population for breastfeeding initiation and breastfeeding duration at 3, 6, and 12 months, overall and by socio-demographic subgroups.

**Table 1:** Unweighted sample sizes and frequencies among US children aged 0 to 24 months from NHAHES, 1999 to 2018

Characteristics	1999–2000	2001–2002	2003–2004	2005–2006	2007–2008	2009–2010	2011–2012	2013–2014	2015–2016	2017–2018	Total
Breastfeeding initiation											
All	755 (9.90%)	939 (12.31%)	849 (11.13%)	908 (11.90%)	811 (10.63%)	780 (10.22%)	625 (8.19%)	673 (8.82%)	695 (9.11%)	594 (7.79%)	7629
Race and ethnicity											
White (Non-Hispanic)	194 (25.70%)	322 (34.29%)	271 (31.92%)	280 (30.84%)	269 (33.17%)	263 (33.72%)	156 (24.96%)	221 (32.84%)	235 (33.81%)	236 (39.73%)	2447 (32.07%)
Black (Non-Hispanic)	125 (16.56%)	218 (23.22%)	206 (24.26%)	182 (20.04%)	135 (16.65%)	122 (15.64%)	157 (25.12%)	139 (20.65%)	125 (17.99%)	112 (18.86%)	1521 (19.94%)
Hispanic	392 (51.92%)	338 (36.00%)	326 (38.40%)	392 (43.17%)	361 (44.51%)	352 (45.13%)	223 (35.68%)	222 (32.99%)	246 (35.40%)	161 (27.10%)	3013 (39.49%)
Other	44 (5.83%)	61 (6.50%)	46 (5.42%)	54 (5.95%)	46 (5.67%)	43 (5.51%)	89 (14.24%)	91 (13.52%)	89 (12.81%)	85 (14.31%)	648 (8.49%)
Poverty index ratio (PIR) ^a^											
Low income (< 1.30)	369 (55.66%)	455 (51.59%)	453 (56.34%)	432 (50.41%)	377 (50.95%)	388 (53.81%)	269 (47.03%)	303 (49.27%)	280 (43.89%)	224 (43.08%)	3550 (50.63%)
High income (≥ 1.30)	294 (44.34%)	427 (48.41%)	351 (43.66%)	425 (49.59%)	363 (49.05%)	333 (46.19%)	303 (52.97%)	312 (50.73%)	358 (56.11%)	296 (56.92%)	3462 (49.37%)
Maternal age at delivery ^a^											
≤ 25 years	381 (50.53%)	470 (50.05%)	413 (48.65%)	418 (46.04%)	357 (44.02%)	321 (41.15%)	246 (39.36%)	272 (40.54%)	227 (32.76%)	219 (36.93%)	3324 (43.60%)
> 25 years	373 (49.47%)	469 (49.95%)	436 (51.35%)	490 (53.96%)	454 (55.98%)	459 (58.85%)	379 (60.64%)	399 (59.46%)	466 (67.24%)	374 (63.07%)	4299 (56.40%)
Smoking status during pregnancy ^a^											
Smoker	105 (13.93%)	125 (13.31%)	138 (16.29%)	99 (10.90%)	113 (13.95%)	88 (11.28%)	65 (10.42%)	58 (8.62%)	66 (9.51%)	81 (13.71%)	938 (12.31%)
Non-smoker	649 (86.07%)	814 (86.69%)	709 (83.71%)	809 (89.10%)	697 (86.05%)	692 (88.72%)	559 (89.58%)	615 (91.38%)	628 (90.49%)	510 (86.29%)	6682 (87.69%)
Breastfeeding duration at 3 months											
All	465 (9.37%)	553 (11.15%)	495 (9.98%)	582 (11.73%)	528 (10.64%)	503 (10.14%)	416 (8.39%)	467 (9.41%)	500 (10.08%)	452 (9.11%)	4961
Race and ethnicity											
White (Non-Hispanic)	118 (25.38%)	194 (35.08%)	163 (32.93%)	189 (32.47%)	169 (32.01%)	174 (34.59%)	105 (25.24%)	163 (34.90%)	171 (34.20%)	182 (40.27%)	1628 (32.82%)
Black (Non-Hispanic)	58 (12.47%)	89 (16.09%)	87 (17.58%)	99 (17.01%)	80 (15.15%)	55 (10.93%)	85 (20.43%)	75 (16.06%)	69 (13.80%)	76 (16.81%)	773 (15.58%)
Hispanic	262 (56.34%)	234 (42.31%)	216 (43.64%)	257 (44.16%)	252 (47.73%)	241 (47.91%)	159 (38.22%)	169 (36.19%)	189 (37.80%)	129 (28.54%)	2108 (42.49%)
Other	27 (5.81%)	36 (6.51%)	29 (5.86%)	37 (6.36%)	27 (5.11%)	33 (6.56%)	67 (16.11%)	60 (12.85%)	71 (14.20%)	65 (14.38%)	452 (9.11%)
Poverty index ratio (PIR) ^a^											
Low income (< 1.30)	215 (52.57%)	239 (46.05%)	233 (50.00%)	252 (45.16%)	208 (43.79%)	237 (51.19%)	168 (43.41%)	195 (45.88%)	187 (40.74%)	166 (42.03%)	2100 (46.09%)
High income (≥ 1.30)	194 (47.43%)	280 (53.95%)	233 (50.00%)	306 (54.84%)	267 (56.21%)	226 (48.81%)	219 (56.59%)	230 (54.12%)	272 (59.26%)	229 (57.97%)	2456 (53.91%)
Maternal age at delivery ^a^											
≤ 25 years	210 (45.16%)	249 (45.03%)	213 (43.03%)	232 (39.86%)	224 (42.42%)	191 (37.97%)	145 (34.86%)	184 (39.40%)	160 (32.06%)	163 (36.06%)	1971 (39.74%)
> 25 years	255 (54.84%)	304 (54.97%)	282 (56.97%)	350 (60.14%)	304 (57.58%)	312 (62.03%)	271 (65.14%)	283 (60.60%)	339 (67.94%)	289 (63.94%)	2989 (60.26%)
Smoking status during pregnancy ^a^											
Smoker	41 (8.82%)	55 (9.95%)	58 (11.72%)	48 (8.25%)	52 (9.85%)	38 (7.55%)	33 (7.95%)	28 (6.00%)	42 (8.42%)	47 (10.42%)	442 (8.91%)
Non-smoker	424 (91.18%)	498 (90.05%)	437 (88.28%)	534 (91.75%)	476 (90.15%)	465 (92.45%)	382 (92.05%)	439 (94.00%)	457 (91.58%)	404 (89.58%)	4516 (91.09%)
Breastfeeding duration at 6 months											
All	432 (9.53%)	497 (10.96%)	457 (10.08%)	527 (11.62%)	480 (10.59%)	471 (10.39%)	381 (8.40%)	430 (9.48%)	457 (10.08%)	402 (8.87%)	4534
Race and ethnicity											
White (Non-Hispanic)	112 (25.93%)	167 (33.60%)	150 (32.82%)	175 (33.21%)	153 (31.88%)	163 (34.61%)	91 (23.88%)	147 (34.19%)	151 (33.04%)	161 (40.05%)	1470 (32.42%)
Black (Non-Hispanic)	56 (12.96%)	84 (16.90%)	84 (18.38%)	90 (17.08%)	77 (16.04%)	53 (11.25%)	82 (21.52%)	73 (16.98%)	65 (14.22%)	71 (17.66%)	735 (16.21%)
Hispanic	240 (55.56%)	213 (42.86%)	196 (42.89%)	229 (43.45%)	224 (46.67%)	226 (47.98%)	146 (38.32%)	155 (36.05%)	173 (37.86%)	111 (27.61%)	1913 (42.19%)
Other	24 (5.56%)	33 (6.64%)	27 (5.91%)	33 (6.26%)	26 (5.42%)	29 (6.16%)	62 (16.27%)	55 (12.79%)	68 (14.88%)	59 (14.68%)	416 (9.18%)
Poverty index ratio (PIR) ^a^											
Low income (< 1.30)	197 (51.98%)	216 (46.45%)	213 (49.42%)	219 (43.37%)	198 (46.05%)	225 (51.49%)	158 (44.76%)	185 (47.19%)	179 (42.72%)	144 (41.26%)	1934 (46.49%)
High income (≥ 1.30)	182 (48.02%)	249 (53.55%)	218 (50.58%)	286 (56.63%)	232 (53.95%)	212 (48.51%)	195 (55.24%)	207 (52.81%)	240 (57.28%)	205 (58.74%)	2226 (53.51%)
Maternal age at delivery											
≤ 25 years	200 (46.30%)	228 (45.88%)	199 (43.54%)	219 (41.56%)	213 (44.38%)	182 (38.64%)	139 (36.48%)	174 (40.47%)	151 (33.04%)	152 (37.81%)	1857 (40.96%)
> 25 years	232 (53.70%)	269 (54.12%)	258 (56.46%)	308 (58.44%)	267 (55.63%)	289 (61.36%)	242 (63.52%)	256 (59.53%)	306 (66.96%)	250 (62.19%)	2677 (59.04%)
Smoking status during pregnancy ^a^											
Smoker	39 (9.03%)	52 (10.46%)	52 (11.38%)	44 (8.35%)	50 (10.42%)	36 (7.64%)	33 (8.68%)	27 (6.28%)	39 (8.55%)	42 (10.47%)	414 (9.14%)
Non-smoker	393 (90.97%)	445 (89.54%)	405 (88.62%)	483 (91.65%)	430 (89.58%)	435 (92.36%)	347 (91.32%)	403 (93.72%)	417 (91.45%)	359 (89.53%)	4117 (90.86%)
Breastfeeding duration at least 12 months											
All	381 (9.52%)	429 (10.72%)	415 (10.37%)	455 (11.37%)	431 (10.77%)	431 (10.77%)	342 (8.54%)	377 (9.42%)	393 (9.82%)	349 (8.72%)	4003
Race and ethnicity											
White (Non-Hispanic)	96 (25.20%)	143 (33.33%)	140 (33.73%)	150 (32.97%)	134 (31.09%)	149 (34.57%)	78 (22.81%)	128 (33.95%)	130 (33.08%)	138 (39.54%)	1286 (32.13%)
Black (Non-Hispanic)	52 (13.65%)	75 (17.48%)	78 (18.80%)	80 (17.58%)	72 (16.71%)	49 (11.37%)	76 (22.22%)	67 (17.77%)	60 (15.27%)	65 (18.62%)	674 (16.84%)
Hispanic	212 (55.64%)	185 (43.12%)	175 (42.17%)	196 (43.08%)	203 (47.10%)	205 (47.56%)	131 (38.30%)	137 (36.34%)	146 (37.15%)	91 (26.07%)	1681 (41.99%)
Other	21 (5.51%)	26 (6.06%)	22 (5.30%)	29 (6.37%)	22 (5.10%)	28 (6.50%)	57 (16.67%)	45 (11.94%)	57 (14.50%)	55 (15.76%)	362 (9.04%)
Poverty index ratio (PIR) ^a^											
Low income (< 1.30)	176 (52.54%)	188 (47.00%)	193 (49.23%)	195 (44.83%)	179 (46.37%)	208 (52.13%)	147 (45.94%)	170 (49.13%)	161 (44.48%)	122 (40.94%)	1739 (47.35%)
High income (≥ 1.30)	159 (47.46%)	212 (53.00%)	199 (50.77%)	240 (55.17%)	207 (53.63%)	191 (47.87%)	173 (54.06%)	176 (50.87%)	201 (55.52%)	176 (59.06%)	1934 (52.65%)
Maternal age at delivery											
≤ 25 years	185 (48.56%)	202 (47.09%)	184 (44.34%)	197 (43.30%)	199 (46.17%)	174 (40.37%)	131 (38.30%)	159 (42.18%)	131 (33.33%)	138 (39.54%)	1700 (42.47%)
> 25 years	196 (51.44%)	227 (52.91%)	231 (55.66%)	258 (56.70%)	232 (53.83%)	257 (59.63%)	211 (61.70%)	218 (57.82%)	262 (66.67%)	211 (60.46%)	2303 (57.53%)
Smoking status during pregnancy ^a^											
Smoker	37 (9.71%)	52 (12.12%)	50 (12.05%)	41 (9.01%)	49 (11.37%)	36 (8.35%)	32 (9.38%)	26 (6.90%)	37 (9.44%)	37 (10.63%)	397 (9.93%)
Non-smoker	344 (90.29%)	377 (87.88%)	365 (87.95%)	414 (90.99%)	382 (88.63%)	395 (91.65%)	309 (90.62%)	351 (93.10%)	355 (90.56%)	311 (89.37%)	3603 (90.08%)

^a^ Numbers vary slightly in the subgroups due to missing data

**Fig. 1:**
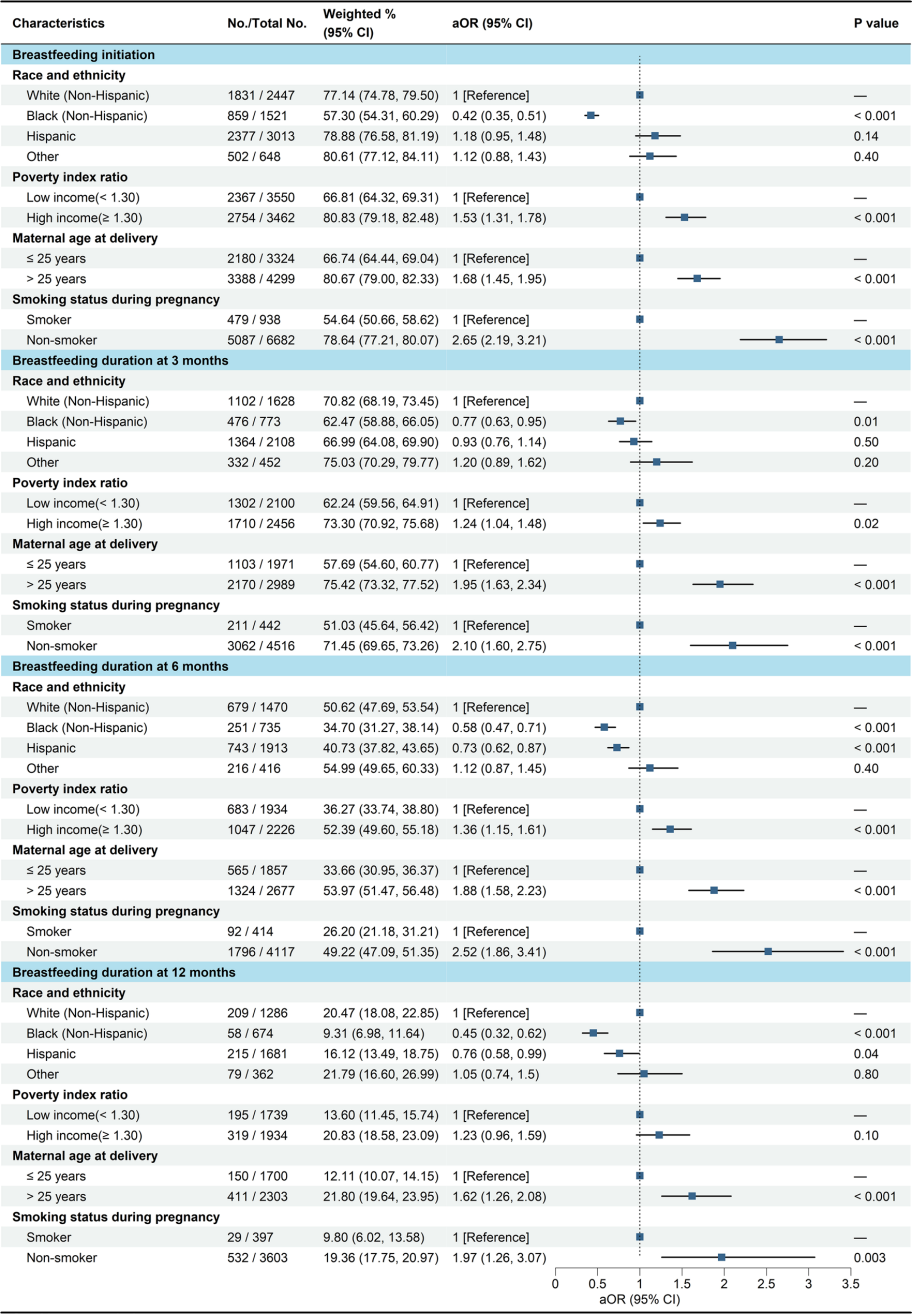
Weighted prevalence and adjusted odds ratios of breastfeeding initiation and breastfeeding duration at 3, 6, and 12 months by socio-demographic characteristics, using binary logistic regression, NHANES 1999 to 2018

Adjusted OR (aOR) and 95% confidence interval (CI) derived from binary logistic regression investigating the odds of breastfeeding initiation and duration at 3, 6, and 12 months with socio-demographic factors were presented in Fig. 1. The prevalence of breastfeeding initiation was lower among non-Hispanic Black children (aOR = 0.42; 95% CI, 0.35, 0.51) when compared with White children, while higher among high-income families (aOR = 1.53; 95% CI, 1.31, 1.78), mothers older than 25 years at delivery (aOR = 1.68; 95% CI, 1.45, 1.95), and nonsmoking mothers during pregnancy (aOR = 2.65; 95% CI, 2.19, 3.21) than among low-income families, mothers aged ≤ 25 years, and smoking mothers, respectively. Consistent with breastfeeding initiation, the prevalence of breastfeeding duration at 3 months was lower among non-Hispanic Black children (aOR = 0.77; 95% CI, 0.63, 0.95), while higher among high-income families (aOR = 1.24; 95% CI, 1.04, 1.48), mothers older than 25 years at delivery (aOR = 1.95; 95% CI, 1.63, 2.34), and nonsmoking mothers during pregnancy (aOR = 2.10; 95% CI, 1.60, 2.75). Similar patterns were observed for the prevalence of breastfeeding duration at 6 and 12 months among these subgroups.

**Fig. 2:**
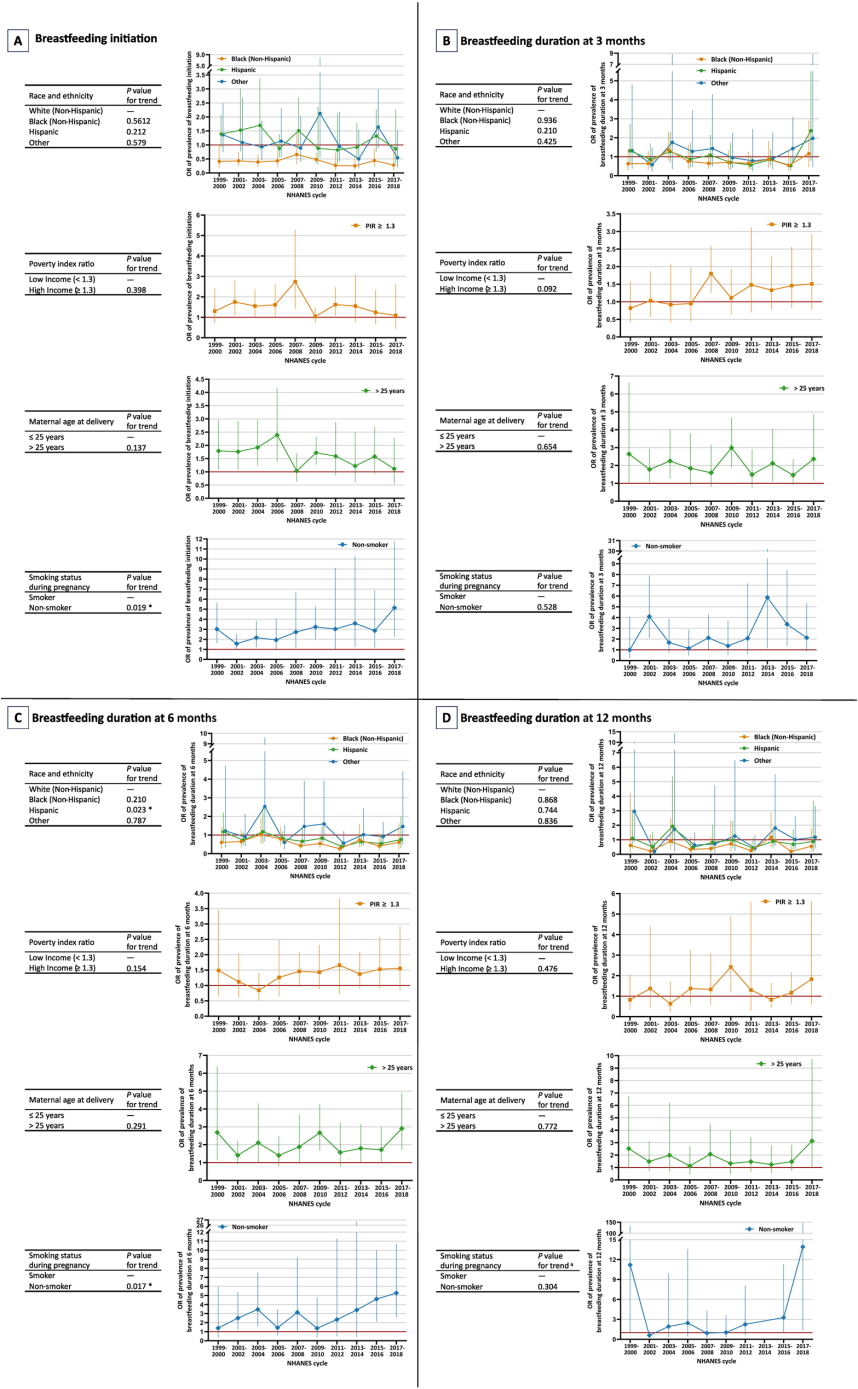
Odds ratios disparity in temporal trends of breastfeeding initiation and duration at 3, 6, and 12 months using meta-regression, NHANES 1999 to 2018. ^a^ The OR of prevalence of breastfeeding duration at 12 months in 2013–2014 was identified as an outlier and excluded from the analysis and the line graph, in order to accurately represent the trend of the dataset

When stratified by 10 consecutive 2-year cycles, we estimated the odds ratios disparity in temporal trends of breastfeeding initiation and duration at 3, 6, and 12 months using meta-regression (Fig. 2). The disparities in prevalence of breastfeeding initiation (β = 0.08; 95% CI, 0.01, 0.15; *p* = 0.019 for trend) and breastfeeding duration at 6 months (β = 0.10; 95% CI, 0.02, 0.19; *p* = 0.017 for trend) became larger over time between smoking and nonsmoking mothers during pregnancy. However, the disparity in prevalence of breastfeeding duration at 6 months narrowed over time between non-Hispanic White and Hispanic children (β = -0.07; 95% CI, -0.14, -0.01; *p* = 0.024 for trend).

**Fig. 3:**
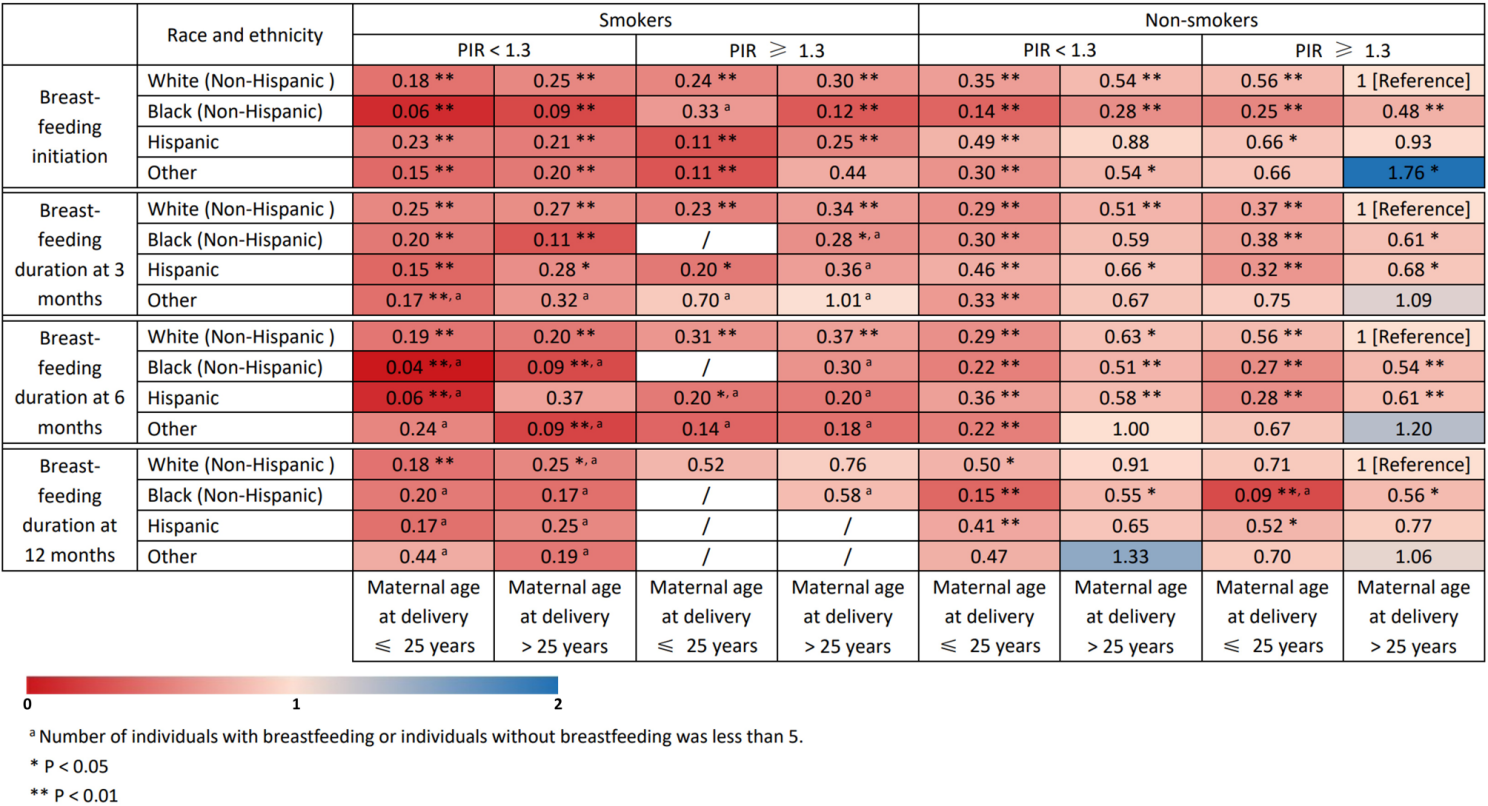
Heat map for the associations of race and ethnicity, poverty index ratio, maternal age at delivery, and smoking status during pregnancy with prevalence of breastfeeding initiation and breastfeeding duration at 3, 6, and 12 months, NHANES 1999 to 2018

Figure 3 summarized a heat map illustrating the combined associations of these significant socio-demographic factors with breastfeeding initiation and breastfeeding duration at 3, 6, and 12 months. Red denoted low prevalence of breastfeeding initiation and breastfeeding duration, and blue denoted high prevalence of breastfeeding initiation and breastfeeding duration. Compared with children born to non-Hispanic White mothers, nonsmoking mothers during pregnancy, mothers older than 25 years at delivery, and high-income families, children born to Black mothers, smoking mothers, mothers aged ≤ 25 years, and low-income families generally had lower odds of breastfeeding initiation and breastfeeding duration at 6 and 12 months. For example, a child born to White mothers, smoking mothers, mothers older than 25 years, and families with low income, quitting smoking during pregnancy would be associated with an additional 116% increase in prevalence of breastfeeding initiation [0.54 − 0.25)/0.25 = 116%]. A child born to Black mothers, nonsmoking mothers, mothers aged ≤ 25 years, and low-income families could expect to have an additional 27% increase in prevalence of breastfeeding duration at 3 months by increasing family income to high [(0.38 − 0.30)/0.30 = 27%].

## Discussion

In this study, we examined the individual and combined associations between socio-demographic factors and breastfeeding rates to identify potential factors that act as barriers or facilitators to breastfeeding practices, using a nationally representative sample of US children. Children born to Black mothers, smoking mothers, mothers aged ≤ 25 years, and low-income families generally exhibited lower odds of breastfeeding initiation and duration at 3, 6, and 12 months. Moreover, the gaps in temporal trends among smoking status even widened.

Smoking during pregnancy was found to be the strongest and most consistent factor associated with lower odds of breastfeeding initiation and duration in our study. Furthermore, the disparities between smoking and nonsmoking mothers became larger over time. Approximately 6.9% of US women smoke during pregnancy [18], suggesting an efficient potential target for increased breastfeeding interventions. Smoking during pregnancy and breastfeeding has been shown to reduce milk production and shorten lactation periods [19]. It is generally accepted that nicotine in mother’s blood stream reduces the level of prolactin, the hormone closely associated with lactation. Moreover, smoke is associated with a lower content of macronutrients, decreased antioxidant properties and altered immune status in human milk [20]. In addition to women smoking throughout pregnancy, 50%–80% of women who quit during pregnancy will relapse to smoking within the first 6 months after birth [21]. However, even if mothers are unable to quit smoking, breastfeeding remains the best choice they can make, as its benefits generally outweigh the risks associated with nicotine exposure [22]. Smoking may be serving, at least in part, as a surrogate measure for socio-economic status [23] and associated challenges to breastfeeding, but nonetheless, its strong association with poorer breastfeeding practices and larger disparity over time among smoking status indicates that increasing the frequency and duration of breastfeeding for smoking mothers should be recognized as a national priority.

Despite differences in how income levels are assessed across countries, families with high income were consistently more likely to initiate and continue breastfeeding. Our study findings further support this observation, with high-income families acting as facilitators of breastfeeding practices. For example, the breastfeeding initiation rate for low-income families was only 66.8%, while it reached 80.8% for high-income families. Working breastfeeding women face conflict in balancing the ability to breastfeed with the demands of work (e.g., inflexible hours and decreased income). Many low-income jobs are not covered under the Family and Medical Leave Act, forcing low-income women to return to work sooner than other women and possibly before breastfeeding is well established [24]. The majority of women in the US return to work between 3 and 6 months after birth, while Black women typically return to work 2 weeks earlier and are more likely to have jobs that are not welcoming to breastfeeding [25]. On the other hand, high-income mothers may have more control over their schedule or work environment, which may provide the support needed to breastfeed for a longer time [8]. It is important to be sensitive to the challenges faced by working women and to take proactive steps to support them. This includes providing information on the benefits of breastfeeding, implementing workplace policies that facilitate breastfeeding, and offering practical strategies and resources that empower women to successfully continue breastfeeding [26].

Although breastfeeding rates in the US have overall risen for several decades, national prevalence data consistently report that Black women have the lowest breastfeeding rates than all other racial/ethnic groups [9, 27]. Our results further confirm this finding, showing that Black mothers typically have the lowest rates of both breastfeeding initiation and duration. Systemic and structural barriers, such as racism, inequitable access to lactation support and resources, and inadequate diversity among the lactation workforce are probably the major causes and drivers of these disparities and inequities [28]. For example, studies from the Black Women’s Health Study cohort have shown that experiences of institutionalized racism in the workplace and growing up in a segregated neighborhood primarily populated by Black residents are associated with both lower rates of breastfeeding initiation and shorter breastfeeding duration [29]. Additionally, health care providers are less likely to discuss breastfeeding options or services with Black women and maternity care practices supportive of breastfeeding are limited in Black communities [30]. Attitudes and subjective norms also may be equally important contributors to the disparities. When asked about historical implications of breastfeeding, Black mothers report that forced wet-nursing by slaves is symbolic of the lack of choice they experienced, in contrast to mother’s right to choose formula feeding [31]. Consequently, formula feeding immediately after birth is nine times higher among Black than White infants [32].

Maternal age is a known limiting factor for breastfeeding initiation and duration, with younger mothers encountering obstacles that impede their access to essential lactation assistance and overall support systems [4]. As our research findings indicate, mothers aged 25 years or younger generally have a lower prevalence of breastfeeding initiation and duration. Older women may be in better circumstances, have higher education, be more financially secure, and may have prior breastfeeding experience, while younger mothers may be less knowledgeable about breastfeeding [33, 34]. For example, in an analysis of data from *Listening to Mothers III*, a national survey including 1598 mothers, breastfeeding supportive practices were differentially implemented by age, with younger mothers reporting less lactation help and fewer evidence-based practices such as rooming in [35]. Younger mothers may also encounter challenges related to personal confidence and self-efficacy in breastfeeding, as well as difficulties in balancing the demands of breastfeeding with other responsibilities such as education and employment. However, studies from Australia and Italy found no and even negative associations between maternal age and breastfeeding rates [36, 37].

The primary strength of our study is the use of nationally representative data collected over a 20-year period, allowing for a comprehensive analysis of both individual and combined socio-demographic factors influencing breastfeeding behavior, as well as an examination of how these factors have evolved over time. Our study has several limitations. Firstly, this study provides evidence on the socio-demographic disparities in breastfeeding practices, but does not provide rationales for these disparities. Additional informative research is needed to understand the basis of low breastfeeding rates and large differentials especially among smoking status and family income. Secondly, the data sources focus on children and do not collect all of the relevant maternal variables, such as education and marital status, which were associated with breastfeeding outcomes. The NHANES is not designed with the main purpose of evaluating breastfeeding practices, and should not be expected to include all variables potentially related to breastfeeding. Thirdly, the data only covers the period up to 2018, which means that any changes in breastfeeding practices and relevant socio-demographic factors that have occurred since then may not be captured, thus limiting the applicability of our findings to the most current context. Finally, the limited sample size, particularly among smoking subgroups in the heat map analysis, restricts our ability to fully understand the extent to which these findings accurately reflect the influence of smoking on breastfeeding outcome.

## Conclusions

In summary, the prevalence of breastfeeding initiation and duration at 3, 6, and 12 months were generally lower among non-Hispanic Black children, low-income families, mothers aged ≤ 25 years, and smoking mothers. These socio-demographic factors serve as significant barriers to their breastfeeding practices. This study provides baseline statistics of socio-demographic disparities on breastfeeding practices, which may be useful for evaluating breastfeeding programs, planning future breastfeeding goals, and prioritizing public health strategies. Given that the disparities among smoking status became larger over time, smoking mothers need to be particularly targeted for promotion and support of breastfeeding.

## Data Availability

Dr. Yongjun Zhang had full access to all the data in the study and takes responsibility for the integrity of the data and the accuracy of the data analysis. Data sharing will be available from Dr. Yongjun Zhang upon a reasonable request.

## Abbreviations

aOR: Adjusted OR
AAP: American Academy of Pediatrics
CI: Confidence intervals
NHAHES: National Health and Nutrition Examination Survey
OR: Odds ratios
PIR: Poverty index ratio
SE: Standard errors
SP: Survey participant
WHO: World Health Organization
